# A Novel sLRP6E1E2 Inhibits Canonical Wnt Signaling, Epithelial-to-Mesenchymal Transition, and Induces Mitochondria-Dependent Apoptosis in Lung Cancer

**DOI:** 10.1371/journal.pone.0036520

**Published:** 2012-05-14

**Authors:** Jung-Sun Lee, Man-Wook Hur, Seong Kyung Lee, Won-Il Choi, Young-Guen Kwon, Chae-Ok Yun

**Affiliations:** 1 Brain Korea 21 Project for Medical Sciences, Yonsei Cancer Center, Yonsei University College of Medicine, Seoul, Korea; 2 Institute for Cancer Research, Yonsei Cancer Center, Yonsei University College of Medicine, Seoul, Korea; 3 Department of Biochemistry and Molecular Biology, Yonsei University College of Medicine, Seoul, Korea; 4 Department of Biochemistry and Molecular Biology, College of Life Science and Biotechnology, Yonsei University, Seoul, Korea; 5 Department of Bioengineering, College of Engineering, Hanyang University, Seoul, Korea; National Taiwan University Hospital, Taiwan

## Abstract

Aberrant activation of the Wnt pathway contributes to human cancer progression. Antagonists that interfere with Wnt ligand/receptor interactions can be useful in cancer treatments. In this study, we evaluated the therapeutic potential of a soluble Wnt receptor decoy in cancer gene therapy. We designed a Wnt antagonist sLRP6E1E2, and generated a replication-incompetent adenovirus (Ad), dE1-k35/sLRP6E1E2, and a replication-competent oncolytic Ad, RdB-k35/sLRP6E1E2, both expressing sLRP6E1E2. sLRP6E1E2 prevented Wnt-mediated stabilization of cytoplasmic β-catenin, decreased Wnt/β-catenin signaling and cell proliferation via the mitogen-activated protein kinase, and phosphatidylinositol 3-kinase pathways. sLRP6E1E2 induced apoptosis, cytochrome *c* release, and increased cleavage of PARP and caspase-3. sLRP6E1E2 suppressed growth of the human lung tumor xenograft, and reduced motility and invasion of cancer cells. In addition, sLRP6E1E2 upregulated expression of epithelial marker genes, while sLRP6E1E2 downregulated mesenchymal marker genes. Taken together, sLRP6E1E2, by inhibiting interaction between Wnt and its receptor, suppressed Wnt-induced cell proliferation and epithelial-to-mesenchymal transition.

## Introduction

Lung cancer is highly aggressive and the most common cause of cancer-related deaths worldwide. In 2009, the American Cancer Society estimated that there were 219,440 new cases of lung cancer in the United States. Standard therapies such as surgery and radiation are not effective in many cases [1]; however, an increased understanding of the molecular mechanisms of lung cancer has led to the development of promising new therapies [2]. Although chemotherapy advances have improved overall survival for patients with aggressive non-small cell lung cancer, chemoresistance remains a major cause of treatment failure [3]. Many aggressive lung cancers show alterations in various cancer-associated genes, including Wnt, K-ras, extracellular signal-regulated kinase (ERK), Akt, and cyclooxygenase-2, suggesting a different molecular pathway for carcinogenesis in lung adenocarcinomas [4]–[6].

The role of Wnt signaling in cancer was first suggested 20 years ago with the discovery of Wnt-1 as an integration site for mouse mammary tumor virus [7]. Many studies have reported that altered expression of Wnt ligands, receptors, and extracellular antagonists are associated with cancer development/progression and stem cell self-renewal/differentiation [8]. Expression of the Wnt ligand, low-density lipoprotein receptor–related protein 5 (LRP5), and LRP6 are upregulated in lung cancers, whereas Wnt antagonists that bind Wnt ligands to block interaction with receptors (e.g., Wnt inhibitory factor-1 (WIF-1), secreted Frizzled-related proteins (sFRP) and dickkopf proteins (DKK) are downregulated or inactivated [9], [10]. Accordingly, monoclonal antibodies and small interfering RNAs against Wnt and overexpression of Wnt antagonists suppress tumor growth in various *in vitro* and *in vivo* tumor models.

LRP6, a member of the LRP superfamily, is required for activation of the canonical Wnt signaling pathway, which leads to the stabilization and nuclear translocation of β-catenin, the key effector molecule [11]. LRP6 consists of four distinct YWTD β-propeller/EGF-like domain pairs; the first and second YWTD domains (E1 and E2) are required for binding to Wnt [12]–[14]. In the present study, we explored the therapeutic potential of a novel soluble Wnt receptor, sLRP6E1E2, which is composed of the LRP6 E1 and E2 regions. We examined the biological effects of sLRP6E1E2 binding to extracellular Wnt ligands and blocking ligand-receptor interactions. Our results provide direct evidence that specific Wnt ligand/receptor interactions have potential use as anticancer therapeutic agents.

## Materials and Methods

### Ethics Statement

Animal handling was conducted in accordance with national and international guidelines, in an animal facility accredited by the Association for Assessment and Accreditation of Laboratory Animal Care (AAALAC). The number of animals used was minimized, and all necessary precautions were taken to mitigate pain or suffering. Protocols were approved by the Institutional Animal Care and Use Committee at Yonsei University health system (2010-0160).

### Materials

Polyclonal antibodies against MAPK kinase (MEK1/2), p44/42 mitogen-activated protein kinase (MAPK; Erk1/2), mTOR, phosphatidylinositol 3-kinase (PI3K) and Akt, and monoclonal antibodies against Wnt3a, Dvl2, Axin, glycogen synthase kinase (GSK3-β), poly (ADP-ribose) polymerase (PARP), and cleaved caspase-3 were purchased from Cell Signaling Technology (Beverly, MA). Antibodies against epithelial-to-mesenchymal transition (EMT)-related molecules β-catenin, E-cadherin and vimentin were obtained from Cell Signaling Technology, and antibody against N-cadherin was purchased from eBioscience (San Diego, CA). Antibodies against cyclin D1 (H-295), cytochrome *c* (C-20 for Western blot analysis), and LRP6 (C-10), and protein A/G agarose beads were purchased from Santa Cruz Biotechnology (Santa Cruz, CA). Monoclonal antibody against caspase-3 was from StressGen Biotechnologies (Victoria, BC). Polyclonal antibody against cytochrome *c* (6H2.B4 for Immunohistochemistry) was from BD Pharmingen (San Diego, CA). Alexa Fluor 488-conjugated and Alexa Fluor 568-conjugated anti-rabbit IgG antibodies were obtained from Invitrogen (Carlsbad, CA). DAPI (1 µg/ml), Hoechst 33342, and tetramethylrhodamine isothiocyanate (TRITC)-conjugated phalloidin were from Sigma (St. Louis, MO). Purified Wnt3a protein was purchased from R&D Systems (Minneapolis, MN).

### Cell Lines and Culture Conditions

Non-small cell lung cancer cell lines A549, H460, H358, and H596 were maintained in Dulbecco’s modified high-glucose Eagle’s medium (DMEM; Life Technologies, Grand Island, NY); H322, H2009 and H1299 cell lines were cultured in RPMI 1640 (Life Technologies) medium supplemented with 10% fetal bovine serum, 2 mM L-glutamine, 1 mM sodium pyruvate, 1% MEM nonessential amino acids, penicillin-streptomycin (100 IU/ml), and Hank’s balanced salt solution (Life Technologies). Cells were purchased from the American Type Culture Collection (Manassas, VA) and maintained at 37°C in a humidified chamber at 5% CO_2_.

### Generation of Adenoviral Vectors Expressing Soluble LRP6 Receptor

**Figure 1:**
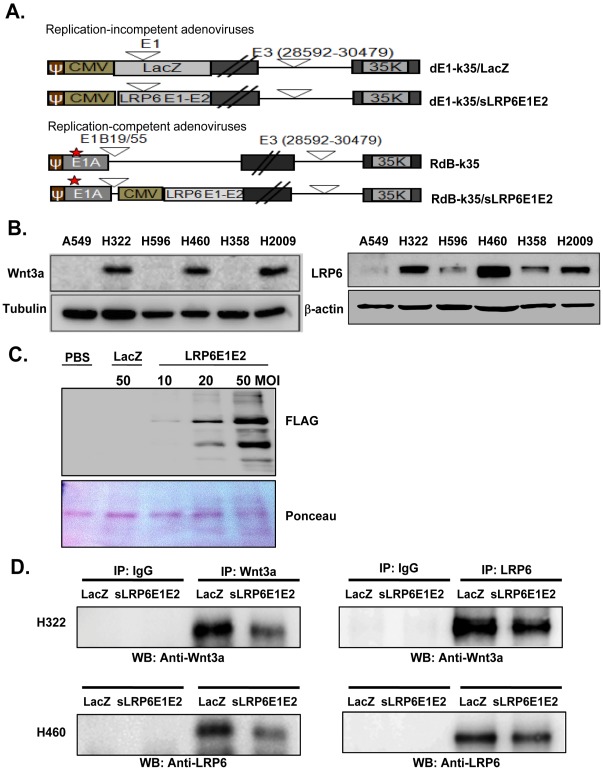
Characterization of the decoy Wnt receptor sLRP6E1E2. (a) Schematic representation of the genomic structure of Ad vectors used. (b) Endogenous Wnt3a (left panel) and LRP6 (right panel) expression in several human lung cancer cell lines. (c) Secretion and expression of sLRP6E1E2. Cell culture supernatants were assessed with FLAG specific Ab (Upper panel). Ponceau staining is shown as loading control (Bottom panel). (d) H322 and H460 cells were transduced with dE1-k35/LacZ or dE1-k35/sLRP6E1E2 (50 MOI) for 48 hr. Cell lysates were immunoprecipitated with antisera against Wnt3a (IP: Wnt3a) or LRP6 (IP: LRP6) followed by western blot analysis with the same antibodies.

To study the biochemical function of soluble LRP6 receptor (sLRP6E1E2), we generated constructs of the E1 and E2 extracellular domains (Wnt-binding sites) of LRP6 [15] and FLAG-tagged sLRP6E1E2 was subcloned into a pCA14 shuttle vector [16]. This pCA14-sLRP6E1E2 vector was co-transformed with a replication-incompetent adenovirus 5/35 chimeric vector (dE1-k35) or replication-competent chimeric oncolytic adenovirus vector (RdB-k35) [17], generating pdE1-k35/sLRP6E1E2 and pRdB-k35/sLRP6E1E2, respectively. These recombinant plasmids were transfected into HEK293 cells to generate dE1-k35/sLRP6E1E2 and RdB-k35/sLRP6E1E2. The replication-incompetent dE1-k35/LacZ and replication-competent oncolytic RdB-k35 vectors were used as negative controls [18] (Fig. 1A). All viruses were obtained as previously described [19].

### Luciferase Reporter Assay for β-catenin Activity

TOPflash and FOPflash luciferase reporter vectors (Upstate Biotechnology, Lake Placid, NY) were used to measure β-catenin/T-cell factor (TCF) signaling activity. A549, H322, and H460 cells were seeded into 6-well plates and transfected with 0.3 µg TOPflash (containing wild-type TCF binding sites) or FOPflash (containing mutated TCF binding sites) negative control with dE1-k35/LacZ or dE1-k35/sLRP6E1E2 (20, 50 MOI) in serum-free medium. After 12 hr, the medium was replaced with 1% DMEM with or without 100 ng/ml of Wnt3a, and the cells were incubated for another 24 hr. Cells were lysed with passive lysis buffer, and 20 µl of the cell extract was analyzed using the Dual-Luciferase Reporter Assay System (Promega, Madison, WI). Experiments were carried out in triplicate and repeated at least three times.

### siRNA Transfection

siRNA transfection was performed as described previously [20]. Briefly, cells were grown in six-well plate to 60% confluence and immediately before transfection washed with serum free medium, and 800 µl of serum-free medium were added per well. Mixture of 0.3 µg TOPflash vector, LRP6-specific or control siRNA (10 nM), and 5 µl of lipofectamine (Invitrogen) in 200 µl of serum-free medium was then incubated for 20 min at room temperature and added into each well. Serum was added 8 hr later to a final concentration of 10%. The next day, cells were stimulated with or without recombinant Wnt3a (100 ng/ml) for an additional 16 hr.

### Cell Proliferation Assay

The cell proliferation assay was determined by 3-(4, 5-dimethylthiazol-2-yl)-2,5-diphenyl-tetrazolium bromide (MTT) assay (Sigma). A549 and H322 cells were seeded in 24-well plates (2×10^4^ cells/well). After 24 hr, cells were treated with PBS, dE1-k35/LacZ, or dE1-k35/sLRP6E1E2. The next day, cells were stimulated with or without recombinant Wnt3a (100 ng/ml) for an additional 48 hr. Absorbance at 540 nm was read on a microplate reader. All assays were performed in triplicate.

### Western Blotting

Cells cultured in DMEM with 1% fetal bovine serum in 100-mm plates were transduced with dE1-k35/LacZ or dE1-k35/sLRP6E1E2. The next day, cells were treated with or without Wnt3a (100 ng/ml) for 16 hr. Immunoblotting was performed as described previously [17]. Blocked membranes were incubated with antibodies against Wnt3a, FLAG, LRP6, Dvl2, Axin, cyclin D1, GSK3-β, MEK1/2, p44/42 MAPK (Erk1/2), Survivin, mTOR, PI3K, Akt, PARP, pro-caspase 3, cleaved-caspase 3, and cytochrome *c* overnight at 4°C. The blots were incubated with the following secondary antibodies conjugated to horseradish peroxidase: goat anti-rabbit IgG, goat anti-mouse IgG, or mouse anti-goat IgG (Cell Signaling Technology) and developed using enhanced chemiluminescence (Amersham Pharmacia Biotech, Uppsala, Sweden).

### Immunoprecipitation Analysis

H322 and H460 cells seeded on 10-cm dishes were infected with each Ad (MOI, 50). Forty-eight hours postinfection, the cells were harvested and lysed in lysis buffer (50 m*M* HEPES containing 0.15 *M* NaCl, 0.5% Nonidet P-40, and proteinase inhibitors phenylmethylsulfonyl fluoride, tosyl-l-lysine chloromethyl ketone, and *N*-tosyl-l-phenylalanine chloromethyl ketone). The total cell lysate (500 µg) was first immunoprecipitated with Wnt3a or LRP6 antibody and analyzed by Western blot with anti-Wnt3a and anti-LRP6 antibody.

### Immunofluorescence Assay

For immunofluorescence microscopy, cultured cells were washed twice with PBS, fixed in 4% paraformaldehyde for 10 min at room temperature, and then permeabilized by incubation for 15 min with 0.1% Triton X-100 in PBS. The samples were blocked with 1% bovine serum albumin followed by incubation with E-cadherin, β-catenin, or anti-cytochrome *c* primary antibodies overnight at 4°C. The next day, cells were washed with PBS and incubated with Alexa Flour 488-conjugated goat anti-rabbit IgG secondary antibody for 60 min at room temperature. The final antibody treatment also contained TRITC-conjugated actin and Hoechst 33342 or DAPI stain (both at 1 µg/ml, Sigma) for nuclear staining. Slides were mounted with Vectashield mounting medium (Vector Laboratories, Burlingame, CA), and cells were viewed under a confocal laser-scanning microscope (LSM510, Carl Zeiss MicroImaging, Thornwood, NY).

### Mitochondrial Fractionation and Western Blotting

Mitochondrial fractions were prepared using the Qproteome mitochondria isolation kit (QIAGEN, Hilden, Germany) following the manufacturer’s instructions. Cells washed with 0.9% sodium chloride solution were suspended with ice-cold lysis buffer by pipetting up and down. After a 10-min incubation, lysate was centrifuged at 1000 *g* for 10 min at 4°C, and the supernatant containing cytosolic proteins was carefully removed. The pellet containing nuclei, cell debris, and unbroken cells was resuspended with ice-cold disruption buffer and centrifuged at 1000 *g* for 10 min at 4°C, and the supernatant (microsomal fraction) was transferred to a clean microtube. The resulting pellet containing mitochondria was washed with the mitochondria storage buffer and centrifuged at 6000 *g* for 20 min at 4°C; a band toward the bottom of the tube was harvested as a mitochondrial fraction. Western blotting was performed with the rabbit anti-cytochrome *c* antibody using the procedure described above.

### Anti-tumor Effects in Human Xenograft Model

Human non-small cell lung cancer (H460) xenograft was established in 6- to 8-week-old male athymic nu/nu mice (Charles River Japan, Yokohama, Japan) by subcutaneous implantation of 1×10^7^ H460 cells in the abdomen. When tumor volumes reached approximately 80–100 mm^3^, the mice were divided five groups with similar mean tumor volumes. Adenoviral vectors were administered intratumorally (2×10^10^ viral particles/mouse) on the first day of treatment (day 1) and days 3 and 5. All animal studies were conducted in the Yonsei University College of Medicine according to institutional regulations, in an animal facility accredited by the Association for Assessment and Accreditation of Laboratory Animal Care (AAALAC). Tumor volume (*V*) was calculated as *V*  = 0.52×*a* ^2^×*b* (*a*, smallest superficial diameter; *b*, largest superficial diameter).

### Tumor Histology and Immunohistochemistry

Tumor tissue was fixed in 4% paraformaldehyde and embedded in paraffin wax for histologic examination and immunohistochemical staining. Representative sections were stained with hematoxylin and eosin and examined by light microscopy. To quantify capillary density and Wnt expression, the tumor sections were stained with anti-mouse CD31 IgG (BD Pharmingen), anti-rabbit β-catenin IgG (Cell Signaling Technology), or anti-mouse Wnt3a IgG (Santa Cruz Biotechnology). After quenching endogenous peroxidase activity and blocking non-specific protein binding with normal goat serum (Vector Laboratories), sections were incubated with primary antibodies at 4°C overnight, and then with biotinylated secondary IgG (Jackson ImmunoResearch, West Grove, PA). Positive immunoreactivity was visualized with ABC-peroxidase kits (ChemMate™ DAKO Envision™ Detection kit; DAKO). Controls were prepared by incubating with irrelevant class-matched and species-matched IgGs. All slides were counterstained with Mayer’s hematoxylin. The expression levels of Wnt3a and β-catenin were assessed semi-quantitatively using MetaMorph® image analysis software (Universal Image Corp., Westchester, PA). Results were expressed as mean optical density for five different digital images.

### Terminal Deoxynucleotidyl Transferase dUTP Nick End Labeling Assay

The 5-µm formalin-fixed and paraffin-embedded tissue sections were deparaffinized and rehydrated according to standard protocols [21]. Apoptosis was detected with the terminal deoxynucleotidyl transferase dUTP nick end labeling (TUNEL) assay (DeadEnd™ Fluorometric TUNEL System; Promega). Briefly, tissue sections were permeabilized with proteinase K (20 µg/ml) for 10 min at room temperature. Sections were then incubated with terminal deoxynucleotidyl transferase (TdT) and fluorescein-12-dUTP in TdT buffer at room temperature for 60 min and washed with TdT buffer. Finally, nuclei were counterstained with DAPI. The samples were analyzed by fluorescence microscopy using a standard fluorescent filter.

### Migration and Invasion Assay

*In vitro* migration assays were performed as described previously [22]. Briefly, the lower surface of 6.5-mm polycarbonate filters (8-µm pore size; Corning Costar, Cambridge, MA) was coated by immersion in 0.1% gelatin. Conditioned media was obtained from A549 cells transduced with PBS, dE1-k35/LacZ and dE1-k35/sLRP6E1E2 after treatment with or without Wnt3a and placed in the bottom Transwell chamber. A549 cells were then plated on the upper chamber (7×10^4^ cells/well). Cultures were incubated at 37°C for 4 hr, fixed, and stained with hematoxylin and eosin. *In vitro* Matrigel invasion assays were performed using bio-coat cell migration chambers. Filters (8-µm pore) were coated with Matrigel basement membrane matrix (37 mg/filter; BD Biosciences, San Jose, CA), and the experiment was performed as described for the cell migration assay. After 24 hr, noninvading cells were removed, and the invading cells on the under surface of the filter were fixed and stained. The membranes were mounted on glass slides, and migrated cells were counted at 200× magnification. Five fields were counted for each assay, and experiments were repeated at least three times.

### Statistical Analysis

Results are expressed as mean ± standard error of the mean (SEM). Group results were compared by one-way analysis of variance, followed by post hoc Student’s *t*-test for unpaired observations or Bonferroni’s correction for multiple comparisons when appropriate. *P*<0.05 was considered significant.

## Results

### Soluble Wnt Decoy Receptor is Expressed in Lung Cancer Cell Lines and Binds to Wnt3a

Endogenous Wnt3a and LRP6 levels were assessed in seven non-small cell lung cancer cell lines (A549, H322, H596, H460, H358, H2009, and H1299) by western blot analysis. Both Wnt3a and LRP6 were more strongly expressed in H322, H460, and H2009 cells than in other cell lines (Fig. 1B and Figure S1); therefore, H322 and H460 cells were selected to evaluate the ability of the soluble Wnt decoy receptor (sLRP6E1E2) to inhibit Wnt signaling. Expression of sLRP6E1E2 from dE1-k35/sLRP6E1E2-transduced A549 cells was confirmed by western blot analysis using anti-FLAG antibodies (Fig. 1C). Secretion of sLRP6E1E2 from dE-k35/sLRP6E1E2-transduced cells was dose-dependent. To ensure equal loading, transferred proteins were visualized by staining with Ponceau Red.

To further investigate if sLRP6E1E2 expressed from dE1-k35/sLRP6E1E2 can interfere the binding ability of endogenous LRP6 to Wnt3a, cell lysates of dE1-k35/LacZ- or dE1-k35/sLRP6E1E2-transduced H322 and H460 cells which endogenously overexpress Wnt3a were immunoprecipitated with Wnt3a or LRP6 antibody, and then endogeneous Wnt3a (top) and total LRP6 (bottom) levels were detected with anti-Wnt3a and anti-LRP6 antibody. We observed that both Wnt3a and LRP6 protein levels were lower in cells transduced with dE1-k35/sLRP6E1E2 than in cells transduced with dE1-k35/LacZ (Fig. 1D), demonstrating that exogenously expressed sLRP6E1E2 can efficiently bind to Wnt3a, leading to prevention of the interaction between endogenous LRP6 and Wnt3a.

### Decoy Wnt Receptor Decreases Cytosolic β-catenin Level and TCF Transcriptional Activity

**Figure 2:**
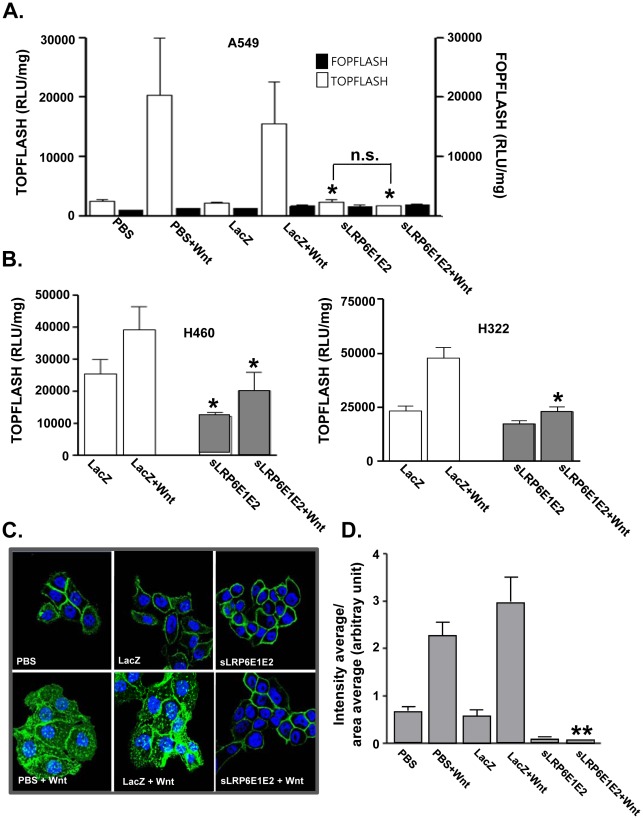
Decoy Wnt receptor sLRP6E1E2 reduces cytosolic β-catenin and T-cell factor transcriptional activity. (a) TCF/LEF luciferase reporter assay in A549 cells. To characterize the sLRP6E1E2 effects on the Wnt3a/β-catenin signaling, cells were transfected with TOPflash (containing wild-type TCF binding sites) or FOPflash (containing mutated TCF binding sites) luciferase vector. *P<0.05 versus dE1-k35/LacZ-transduced or PBS-treated cells. (b) TCF/LEF luciferase reporter assay in H460 and H322 cells. *P<0.05 versus PBS or dE1-k35/LacZ-transduced cells with or without Wnt3a. (c) H322 cells were transduced with dE1-k35/LacZ or dE1-k35/sLRP6E1E2 (50 MOI) with or without Wnt3a. Cells were labeled with anti-β-catenin. Original magnification, ×630. (d) Semi-quantitative analysis of panel (c) results using MetaMorph® imaging analysis software. Each data point indicates mean ± SEM (each group, n = 5). **P<0.001 versus PBS or dE1-k35/LacZ-transduced cells with Wnt3a.

We next hypotheses that secreted sLRP6E1E2 protein inhibit Wnt signaling by direct binding to Wnt. Therefore, to characterize the sLRP6E1E2 effects on the Wnt3a/β-catenin signaling, we determined its effect on β-catenin using a luciferase reporter system activated by β-catenin/TCF [23]. As shown in Fig. 2A, luciferase activity was low in A549 cells transduced with dE1-k35/LacZ or dE1-k35/sLRP6E1E2 in the absence of Wnt3a, since the endogenous expression level of Wnt3a in A549 is very minimal (Fig. 1B). Wnt3a treatment increased luciferase expression approximately 7- to 8-fold in control cells, but not in dE1-k35/sLRP6E1E2-transduced cells, suggesting that secreted sLRP6E1E2 could block the signaling effect of exogenously treated Wnt3a. In the absence of Wnt3a, luciferase activity was reduced by dE1-k35/sLRP6E1E2 in H460 (48%) and H322 (12%) cells compared with dE1-k35/LacZ controls (Fig. 2B; *P*<0.05). Wnt3a stimulation increased luciferase activity in H460 (53%) and H322 (102%) cells transduced with dE1-k35/LacZ, but luciferase activity was significantly lower in dE1-k35/sLRP6E1E2-transduced H460 (48%) and H322 (52%) cells compared with dE1-k35/LacZ (*P*<0.05). In order to make this result more compelling, we investigated the effect of LRP6-specific siRNA (si-LRP6) on the Wnt3a/β-catenin signaling. As shown in Figure S2, luciferase activity was significantly reduced by the treatment of si-LRP6 in both presence and absence of Wnt3a, in agreement with result of above (Fig. 2).

To evaluate the effect of sLRP6E1E2 on β-catenin localization, immunofluorescence staining was performed in H322 cells treated with PBS or transduced with dE1-k35/LacZ or dE1-k35/sLRP6E1E2. In the absence of Wnt3a, β-catenin staining was restricted primarily to cell–cell contact sites in all groups. Upon Wnt3a stimulation, control cells (PBS and dE1-k35/LacZ) showed reduced β-catenin localization at the plasma membrane, especially at cell–cell junctions, and increased β-catenin levels in the cytosol and nucleus. In contrast, dE1-k35/sLRP6E1E2-transduced cells showed lower levels of cytosolic β-catenin, and higher levels of membrane-associated β-catenin (Fig. 2C). Quantification of the nucleus β-catenin expression showed a 98.08% decrease in dE1-k35/sLRP6E1E2-transduced cells compared with dE1-k35/LacZ controls in the presence of Wnt3a (Fig. 2D). Results of these functional studies demonstrate that interactions between sLRP6E1E2 and Wnt may be sufficient to block Wnt signaling.

### Decoy Wnt Receptor sLRP6E1E2 Inhibits Lung Cancer Cell Proliferation

**Figure 3:**
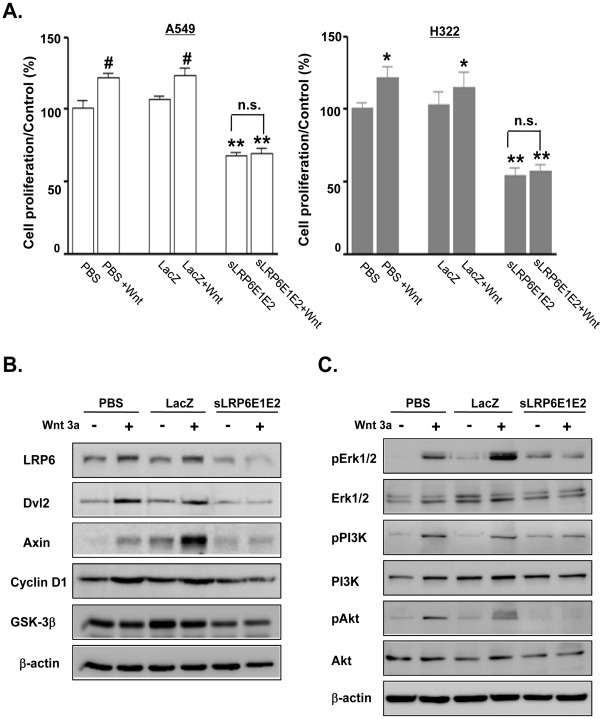
Decoy Wnt receptor sLRP6E1E2 decreases proliferation in human lung cancer cells. (a) A549 and H322 cells were transduced with dE1-k35/LacZ or dE1-k35/sLRP6E1E2 (20 MOI). The next day, these cells were incubated with or without Wnt3a (100 ng/ml). After 3 days, cell proliferation was assessed by the MTT assay (mean ± SEM). *P<0.05, ^#^ *P*<0.01 versus untreated control for each group; **P<0.001 versus dE1-k35/LacZ-transduced or PBS-treated cells. n.s.  =  not significant. (b) A549 cells were treated as indicated above (Fig. 3a). Western blot using antibodies specific to LRP6, Dvl2, Axin, Cyclin D1, or GSK-3β. (c) A549 cells were harvested at 6 hr after Wnt3a treatment. The p-Erk1/2, Erk1/2, p-PI3K, PI3K, p-Akt, and Akt proteins were detected by western blot analysis.

The Wnt pathway regulates a wide range of cellular functions including proliferation [24]. To test the effects of sLRP6E1E2 on proliferation of A549 and H322 cells *in vitro*, cells were treated with PBS or transduced with dE1-k35/LacZ or dE1-k35/sLRP6E1E2. At 72 hr after transduction with dE1-k35/sLRP6E1E2 (20 MOI), cell proliferation was reduced by 39% in A549 cells and 51% in H322 cells compared with dE1-k35/LacZ-transduced controls. Wnt3a stimulation increased proliferation approximately 10–20% in control cells, but had no apparent effect on dE1-k35/sLRP6E1E2-transduced cells. Proliferation was 54% lower in A549 cells and 61% lower in H322 dE1-k35/sLRP6E1E2-transduced cells than dE1-k35/LacZ-transduced cells (*P*<0.001; Fig. 3A).

To characterize signaling pathways involved in the anti-proliferative action of sLRP6E1E2, we examined its effects on canonical Wnt signaling. As shown in Fig. 3B, LRP6, Dvl2 and Axin protein levels in control cells (PBS and dE1-k35/LacZ) were increased by Wnt3a, but were apparently unaltered by Wnt3a in dE1-k35/sLRP6E1E2-transduced cells. Similarly, cyclin D1 expression was slightly increased in control cells following Wnt3a stimulation, but slightly decreased in dE1-k35/sLRP6E1E2-transduced cells. GSK3β levels also appeared slightly decreased after Wnt3a treatment.

Wnt plays a fundamental role in proliferation by activating Erk1/2 and PI3K-Akt pathways [25]. We therefore investigated whether sLRP6E1E2 can downregulate these pathways. As shown in Fig. 3C, phosphorylation of Erk1/2, PI3K, and Akt was upregulated by Wnt3a treatment, but levels of phorphorylation was lower in dE1-k35/sLRP6E1E2-transduced cells compared to those in PBS-treated and dE1-k35/LacZ-transduced cells. Expression of mTOR, PI3K, and Akt was not affected by Wnt3a stimulation, and was lower in dE1-k35/sLRP6E1E2-transduced cells than controls in H460 cells (Figure S3). Taken together, these results suggest that sLRP6E1E2 exerts antiproliferative actions by inhibiting Wnt signaling via MEK-ERK and PI3K- Akt pathways.

### Decoy Wnt Receptor sLRP6E1E2 Induces Apoptosis

**Figure 4:**
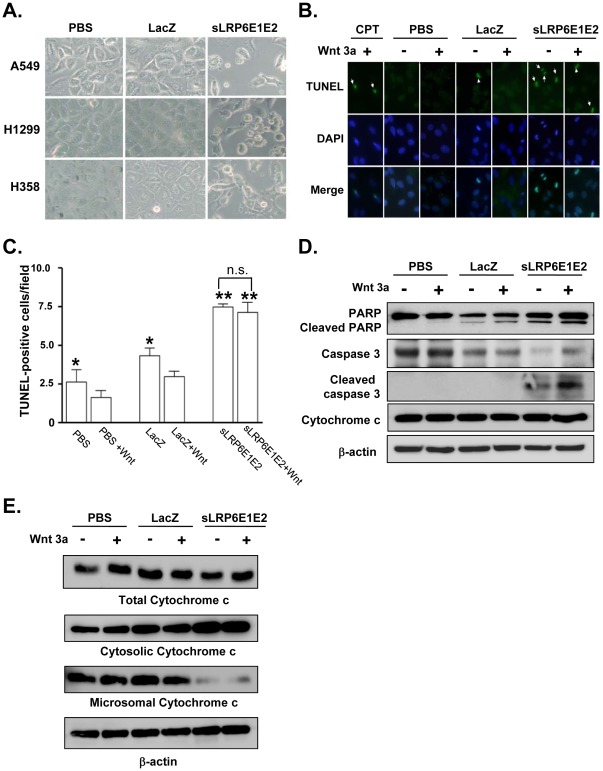
Decoy Wnt receptor sLRP6E1E2 induces apoptosis in human lung cancer cells. (a) Cells were transduced with dE1-k35/LacZ or dE1-k35/sLRP6E1E2 at (20 MOI), and photographs were taken at 72 hr later. Original magnification, ×200. (b) Detection of sLRP6E1E2-induced apoptosis by TUNEL staining. Original magnification, ×400. (c) Total number of TUNEL-positive cells per fields (mean ± SEM). *P<0.05 versus PBS or dE1-k35/LacZ treated with Wnt3a; **P<0.001 versus PBS-treated or dE1-k35/LacZ-transduced controls. n.s.  =  not significant. (d) Western analysis of sLRP6E1E2-mediated apoptosis. H460 cells were transduced with dE1-k35/LacZ or dE1-k35/sLRP6E1E2 (20 MOI). The western blot using specific antibodies against uncleaved PARP, cleaved PARP, pro-caspase-3, cleaved caspase-3, and cytochrome *c*. (e) H460 cells were treated as indicated above (Fig. 4d). Subcellular localization of cytochrome *c* was determined by western blot analysis of cytosolic and microsomal fractions.

Wnt signaling can prevent apoptosis and promote cellular proliferation and survival [26]. To characterize the molecular mechanisms by which sLRP6E1E2 inhibits non-small cell lung cancer proliferation, we evaluated the effects of sLRP6E1E2 on apoptosis. At 3 days after dE1-k35/sLRP6E1E2 transduction, we observed that A549, H1299, and H358 cells gradually detached from the culture dish and became rounder and smaller than attached cells (Fig. 4A), suggesting that sLRP6E1E2 induced apoptosis. Evidence of apoptosis was sought by looking for nuclear apoptotic bodies (data not shown), and then assessed using the TUNEL assay to detect internucleosomal DNA fragmentation [27]. As shown in Fig. 4B, more TUNEL-positive cells were observed among dE1-k35/sLRP6E1E2-transduced cells than among control cells in the presence or absence of Wnt3a. Quantitation of TUNEL staining revealed that the rate of apoptosis was approximately 1.9-fold higher (without Wnt3a) and 2.8-fold higher (with Wnt3a) in dE1-k35/sLRP6E1E2-transduced cells than in dE1-k35/LacZ-transduced controls *(P*<0.001) (Fig. 4C).

We next evaluated regulators of apoptosis, of which the caspase family and cytochrome *c* are the best characterized. In the absence and presence of Wnt3a, full-length 116-kDa PARP protein was reduced and 85-kDa cleavage fragments were increased in dE1-k35/sLRP6E1E2-transduced cells (Fig. 4D). Levels of the cleaved (active) form of caspase-3 were also markedly increased by sLRP6E1E2. As shown in Fig. 4E, dE1-k35/sLRP6E1E2-transduced cells also showed increased cytosolic cytochrome *c* and decreased microsomal cytochrome *c*. Stimulation with Wnt3a produced similar effects.

### Decoy Wnt Receptor sLRP6E1E2 Inhibits Tumor Xenograft Growth

**Figure 5:**
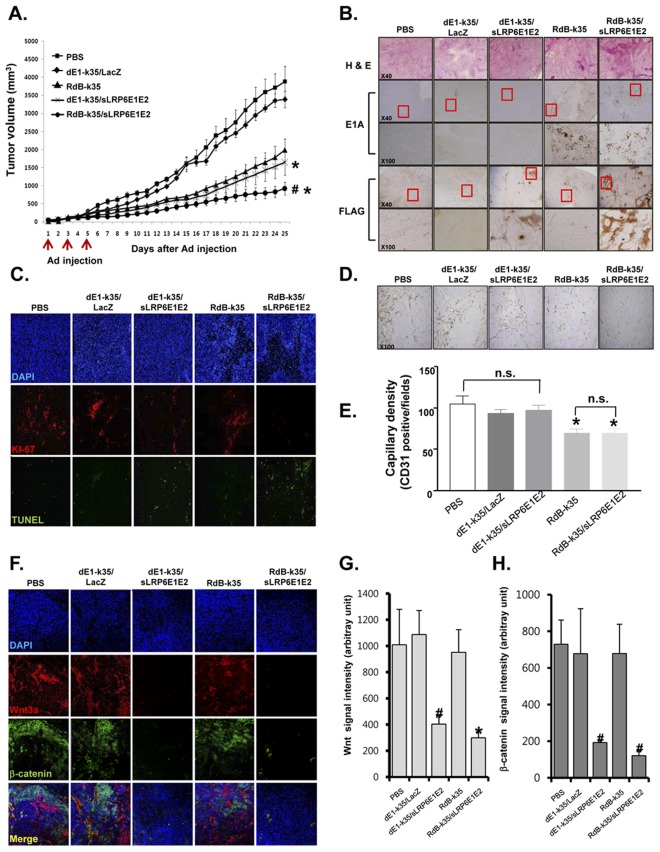
Decoy Wnt receptor sLRP6E1E2 inhibits tumor growth and characterization. (a) Tumors were injected with PBS (▪), dE1-k35/LacZ (♦), RdB-k35 (▴), dE1-k35/sLRP6E1E2 (×), or RdB-k35/sLRP6E1E2 (•) on days 1, 3, and 5. Results are expressed as mean ± SEM (n = 7). *P<0.05 versus PBS-treated or dE1-k35-treated controls and versus dE1-k35/sLRP6E1E2. ^#^ *P*<0.01 versus PBS-treated or dE1-k35-treated controls. (b) Tumor sections from each group were immunostained against E1A or FLAG (original magnification, ×40 and ×100). (c) Tumor tissues from each group were stained with DAPI (blue), anti-Ki67 (red), and TdT-mediated TUNEL (green). Original magnification: ×100. (d) Blood vessels were visualized by staining for CD31. Original magnification, ×100. (e) Mean microvessel density for each treatment group (CD31 positive cells/field). Results are expressed as mean ± SEM (each group, n = 3 tumors). *P<0.05 versus PBS, dE1-k35, or dE1-k35/sLRP6E1E2. n.s.  =  not significant. (f) Cells were stained with DAPI (blue), anti-Wnt3a (red), or anti-β-catenin (green). Original magnification: ×100. (g, h) The expression levels of Wnt3a (g) and β-catenin (h) were assessed semi-quantitatively using MetaMorph® imaging analysis software. Results are expressed as mean ± SEM (each group, n = 5 tumors). #*P*<0.01 versus dE1-k35, *P<0.05 versus RdB-k35.

We next evaluated the ability of sLRP6E1E2 to inhibit tumor growth in a mouse xenograft model. Tumors were generated by subcutaneous injection of H460 cells into the abdominal region of nude mice. When tumors reached a mean size of 80–100 mm^3^, they were injected with PBS, dE1-k35, RdB-k35, dE1-k35/sLRP6E1E2, or RdB-k35/sLRP6E1E2 on days 1, 3, and 5. Fig. 5A shows that the volume of tumors injected with sLRP6E1E2-expressing vectors was significantly lower than that of corresponding controls. After 25 days, tumors treated with PBS reached a mean volume of 3883.1±418.08 mm^3^, and tumors treated with dE1-k35 and RdB-k35 reached 3388.1±226.9 mm^3^ and 1991±311.8 mm^3^, respectively. In contrast, tumor growth was strongly suppressed in mice injected with dE1-k35/sLRP6E1E2 (1645.3±353.6 mm^3^; *P*<0.05 compared with PBS or dE1-k35 groups) or RdB-k35/sLRP6E1E2 (923.3±180.4 mm^3^; *P*<0.01 compared with PBS or RdB-k35 groups).

To evaluate the biological effects of sLRP6E1E2 in tumor tissue, tumors were harvested 3 days after the final adenovirus injection. Analysis of adenoviral E1A protein expression revealed that RdB-k35 and RdB-k35/sLRP6E1E2 had replicated and spread through the tumor (Fig. 5B, E1A). Immunohistochemical analysis of sLRP6E1E2 (Fig. 5B, FLAG) showed that its expression was more widespread in RdB-k35/sLRP6E1E2-treated tumors than in dE1-k35/sLRP6E1E2-treated tumors, indicating that the oncolytic adenovirus more efficiently expressed sLRP6E1E2 than the replication-incompetent adenovirus, contributing to its superior antitumor actions.

### Anti-proliferative and Apoptotic Effects of sLRP6E1E2-expressing Vectors in H460 Xenografts

To assess the effects of sLRP6E1E2 on tumor xenograft growth in mice, tumor samples were analyzed by Ki-67 immunostaining for proliferating cells and TUNEL staining for apoptotic cells. We found that Ki-67 expression was reduced and TUNEL-positive cells were increased in tumors treated with dE1-k35/sLRP6E1E2 or RdB-k35/sLRP6E1E2 compared with corresponding controls (Fig. 5C). We also detected more TUNEL-positive cells in RdB-k35/sLRP6E1E2-treated tumors than in dE1-k35/sLRP6E1E2-treated tumors, consistent with previous results. To determine whether the smaller sLRP6E1E2-treated tumors exhibited reduced neovascularization, microvessel density was assessed by CD31 staining. Fewer endothelial cells and vessel structures was observed in tissues injected with E1-expressing oncolytic adenoviruses (RdB-k35 and RdB-k35/sLRP6E1E2) than PBS-treated tumors (*P*<0.05), whereas no significant decrease in vascular density was observed in tumors injected with dE1-k35 or dE1-k35/sLRP6E1E2 (Fig. 5D & 5E). Further, vessel density in tumors injected with sLRP6E1E2-expressing adenoviruses did not differ from their corresponding controls, suggesting that the antitumor properties of sLRP6E1E2 were not mediated by anti-angiogenic effects.

To further investigate the role of Wnt signaling in the antitumor actions of sLRP6E1E2-expressing adenoviruses, Wnt and β-catenin localization in tumor tissue was evaluated. High endogenous expression of β-catenin and Wnt was observed in tumor tissues treated with PBS or control vectors (dE1-k35 and RdB-k35) (Fig. 5F-H), but was significantly reduced by sLRP6E1E2-expressing vectors, suggesting that blockade of Wnt signaling in tumor cells was an important contributor to slower tumor growth.

### Wnt Treatment Results Altered Cell Morphology and Induces EMT in Tumor Cells

**Figure 6:**
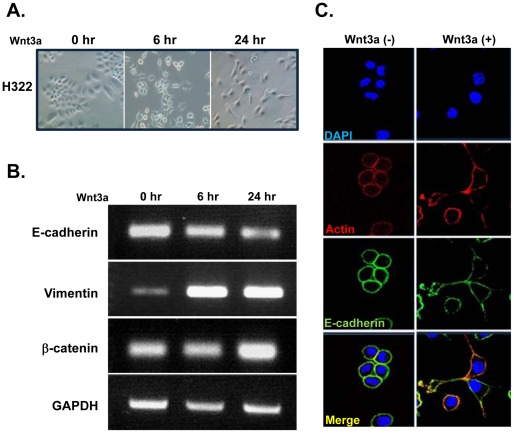
Wnt3a treatment results in the disruption of cell-cell junctions and epithelial-to-mesenchymal transition in tumor cells. (a) H322 cells were treated with Wnt3a (100 ng/ml) for the indicated times, and morphology changes were observed by light microscopy. Original magnification, ×200. (b) E-cadherin, Vimentin, and β-catenin mRNA levels in H322 cells after Wnt3a treatment. (c) H322 cells were stained with DAPI (blue), TRITC-labeled actin (red), or anti E-cadherin (green) after 24 incubation with or without Wnt3a (100 ng/ml). Original magnification, ×630.

EMT is an important process in tumor development, and the Wnt/β-catenin signal pathway may play an important role in this process. Therefore, we investigated whether Wnt3a could induce EMT in H322 cells. We found that cells became elongated and spindle-shaped 1 day after Wnt3a treatment, resembling the morphology of mesenchymal cells (Fig. 6A). We also observed increased expression of mesenchymal markers Vimentin and β-catenin with a concomitant decrease in epithelial marker E-cadherin (Fig. 6B). Immunofluorescence staining revealed that actin and E-cadherin levels were dramatically reduced in cell–cell contacts after Wnt3a treatment (Fig. 6C and Figure S5).

### sLRP6E1E2 Modulates EMT-related Marker Expression and MMP-2/MMP-9 Activity

**Figure 7:**
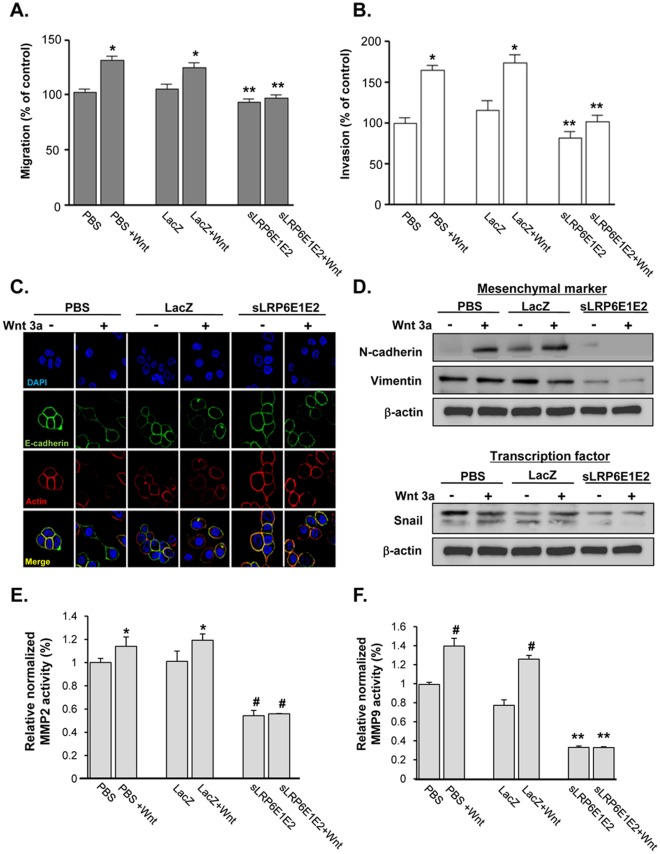
Decoy Wnt receptor sLRP6E1E2 inhibits cancer cell migration and invasion, and modulates expression of epithelial-to-mesenchymal transition markers and MMPs. (a) Quantitative analysis of A549 lung cancer cell migration. Experiments were performed in triplicate, and results are expressed as mean ± SEM. *P<0.05 versus PBS- or dE1-k35/LacZ-treated controls; **P<0.001 versus PBS or dE1-k35/LacZ with Wnt3a. (b) Invasion of tumor cells was quantified as number of cells in five fields of view per filter. Experiments were performed in triplicate, and results are expressed as mean ± SEM. *P<0.05 versus PBS- or dE1-k35/LacZ-treated controls; **P<0.001 versus PBS or dE1-k35/LacZ with Wnt3a. (c) Expression of EMT markers in H322 cells after 24 hr treatment with PBS, dE1-k35/LacZ, or dE1-k35/sLRP6E1E2 in the presence and absence of Wnt3a (100 ng/ml). Cells were stained with DAPI (blue), TRITC-labeled actin (red), or anti E-cadherin (green). Original magnification, ×630. (d) Expression of EMT-related markers in H322 cell lines. Expression levels of mesenchymal markers (N-cadherin & vimentin) as well as transcriptional factor (Snail) was determined by Western blotting. (e, f) A549 cells were transduced with dE1-k35/LacZ or dE1-k35/sLRP6E1E2 with or without Wnt3a (100 ng/ml). The enzyme activity of MMP-2 and MMP-9 was measured in supernatants collected from transduced cells at 48 hr using the Sensolyte 520 MMP-2 and MMP-9 assay kit. Experiments were performed in triplicate, and results are expressed as mean ± SEM. (e) *P<0.05, (f) ^#^ *P*<0.01 versus PBS- or dE1-k35/LacZ-treated controls; (e) ^#^ *P*<0.01, (f) **P<0.001 versus PBS or dE1-k35/LacZ with Wnt3a.

Acquisition of migratory properties by cancer cells is important for metastatic tumor cell spread [28]. Because increasing Wnt3a appeared to enhance motility and invasiveness, we asked whether interfering with the Wnt signaling pathway by expressing sLRP6E1E2 would inhibit *in vitro* motility and invasion. We examined the effect of sLRP6E1E2 on A549 cells using transwell motility and matrigel invasion assays. We collected conditioned medium from PBS-treated, dE1-k35/LacZ-transduced, and dE1-k35/sLRP6E1E2-transduced cells after treatment with or without Wnt3a. Conditioned medium from dE1-k35/sLRP6E1E2-transduced cells inhibited migration by 12.4% (without Wnt3a) and 23.8% (with Wnt3a) compared with conditioned medium from dE1-k35/LacZ-transduced cells (*P*<0.001) (Fig. 7A). Similarly, conditioned medium from dE1-k35/sLRP6E1E2-transduced cells inhibited invasion by 34.2% (without Wnt3a) and 56.2% (with Wnt3a) compared with conditioned medium from dE1-k35/LacZ-transduced cells (Fig. 7B).

EMT has been shown to be important for cancer progression and metastasis. Therefore, we examined whether sLRP6E1E2 can modulate EMT-related markers associated with tumor invasion in H322 cell. Figure 7C and Figure S5 showed that dE1-k35/sLRP6E1E2-transduced cells exhibited up-regulation of epithelial markers E-cadherin and actin by immunofluorescent staining. Conversely, mesenchymal markers (i.e., N-cadherin and vimentin) were markedly down-regulated in dE1-k35/sLRP6E1E2-transduced cells (Fig. 7D, upper panel). The expression of transcription factor Snail which is known to repress E-cadherin and promote a mesenchymal phenotype [29] was also down-regulated (Fig. 7D, lower panel). Together, these data further support the role of sLRP6E1E2 in modulating EMT-related events.

Several MMPs are additional Wnt target genes that play an important role in promoting invasion and metastasis of malignantly transformed cells [30], [31]. We therefore examined the effect of sLRP6E1E2 on expression of MMP-2 and MMP-9, which play a critical role in angiogenesis, tumor growth, and metastasis. As shown in Fig. 7E and F, Wnt3a stimulation upregulated MMP-2 and MMP-9 enzyme activity in PBS-treated and dE1-k35/LacZ-transduced A549 cells, but dE1-k35/sLRP6E1E2-transduced cells showed low MMP-2 and MMP-9 enzyme activity with or without Wnt3a treatment. Taken together, these findings suggest that sLRP6E1E2 affected multiple Wnt-related pathways in human non-small cell lung cancer cell lines, leading to reduced cellular invasiveness.

## Discussion

Aberrant activation of the Wnt pathway contributes to human cancer progression [32]. Accordingly, monoclonal antibodies against Wnt ligands [33], [34] and overexpression of Wnt antagonists [35], [36] are able to reduce *in vivo* tumor growth. Members of the sFRP family bind directly to Wnts, inhibiting their ability to bind to the Wnt receptor complex. The Fzd8 soluble extracellular domain suppresses Wnt-driven tumor growth *in vivo* [10] and two sFRPs, FrzA and FrzB inhibited Wnt-1–mediated increase in cytoplasmic β-catenin levels, TCF transcriptional activity *in vitro*, and tumor growth and metastasis [37].

Antagonists that interfere with Wnt ligand/receptor interactions may therefore be potent cancer treatments. However, primary human tumors and cancer cell lines express multiple Wnt and Fzd receptors, and the specificity of Wnt proteins for the various receptors is unclear [37]. Therefore, it is difficult to design a Wnt antagonist that can block these interactions. Recently, Lu et al. reported that cotransfection of vectors expressing Wnt3 and LRP6 receptor increased TCF activation [38], suggesting the therapeutic potential of a soluble LRP6 receptor as a Wnt antagonist. Therefore, we generated sLRP6E1E2 based on the LRP6 EGF repeats required for functional interaction with Wnt.

In the present study, we demonstrated that sLRP6E1E2 is secreted and binds specifically to Wnt3a, as evidenced by decreased endogenous Wnt3a and LRP6 levels after transduction with sLRP6E1E2-expressing adenoviruses (Fig. 1). Wnt signaling affects multiple targets; therefore, we then assessed the effect of sLRP6E1E2 on pathways responsible for tumor growth, invasion, and metastasis. Our *in vitro* studies showed that sLRP6E1E2 reduced cell proliferation by inhibiting MEK-ERK and PI3K- Akt signaling (Fig. 3A, C and Figure S3). Since PI3K-Akt signaling regulates cell survival and apoptosis [39], the ability of sLRP6E1E2 to induce apoptosis was assessed. As shown in Fig. 4, dE1-k35/sLRP6E1E2 transduction increased cytosolic cytochrome *c* levels, consistent with apoptosis through a mitochondria-dependent pathway.

Limitations of replication-incompetent adenoviruses for cancer therapy include nonselective delivery of therapeutic genes to both normal and tumor cells, and inability to replicate and spread to neighboring tumor cells. To improve the therapeutic value of adenovirus-mediated gene therapy, a cancer cell-specific replicating adenovirus (oncolytic adenovirus) has been developed [40]. Our group previously developed RdB, an E1A-E1B double mutant oncolytic adenovirus with higher cancer cell-specific cytotoxicity and viral replication than E1A or E1B single mutant oncolytic adenoviruses [19]. As shown in Fig. 5, tumors treated with RdB-k35/sLRP6E1E2 were 54% smaller than tumors treated with the oncolytic adenovirus not expressing sLRP6E1E2 (RdB-k35) and 44% smaller than those treated with the non-replicating dE1-k35/sLRP6E1E2. RdB-k35/sLRP6E1E2 increased apoptosis, but also exerted anti-angiogenic effects. Immunostaining tumor tissues against CD31, a marker of angiogenesis, showed that the control oncolytic adenovirus RdB-k35 produced effects similar to that of RdB-k35/sLRP6E1E2. We and other groups previously demonstrated that replication-competent adenoviruses suppress tumor angiogenesis through the preserved E1A region [17], [41], [42], indicating that sLRP6E1E2 expression from the vectors does not play a role in reducing tumor angiogenesis.

During tumor metastasis, disseminated cancer cells appear to require the ability to self-renew, similar to that exhibited by stem cells. Our results show that Wnt signaling upregulates EMT-related molecules Vimentin and β-catenin and increased tumor cell migration and invasion (Fig. 6 and Figure S4). Cells were more compact and adhesive after treatment with the sLRP6E1E2-expressing adenovirus (data not shown), with increased expression of epithelial markers (E-cadherin and actin filaments) and down-regulation of mesenchymal markers (vimentin, N-cadherin, and Snail) (Fig. 7C and D). Moreover, sLRP6E1E2 reduced expression of MMP-2/MMP-9, which correlate with tumorigenicity and metastatic potential of cancer cells [43]. Therefore, it is important to determine whether targeting Wnt ligand-receptor interactions will reduce tumor recurrence and/or metastasis, warranting future investigation.

Many studies have demonstrated the association between aberrant expression of Wnt ligands/receptors and human cancer development/progression. The current study demonstrates for the first time that a decoy receptor consisting of LRP6 Wnt-binding domains can effectively inhibit Wnt signaling and downregulate potential Wnt targets. In addition, sLRP6E1E2 markedly reduced tumor growth, invasion, and EMT. Taken together, our findings demonstrate the therapeutic potential of sLRP6E1E2 as a novel cancer gene therapy. Ongoing studies in our laboratories are aimed at determining the efficacy of sLRP6E1E2 against cancer stem cells.

## Supporting Information

Figure S1 Endogenous LRP6 and Wnt3a expression in HT1299 human lung cancer cells. Western blot using antibodies specific to Wnt3a or LRP6.
(TIF)

Figure S2 The effect of LPR6 knockdown on β-catenin/TCF transcriptional activity. H460 (a) and H322 (b) cells were co-transfected with TOPflash vector and LRP6 siRNA or control siRNA (si-Scramble) in the presence or absence of Wnt3a for 16 hr as described in Materials and Methods. **P<0.001 versus si-Scramble-transfected cells with or without Wnt3a.
(TIF)

Figure S3 Decoy Wnt receptor sLRP6E1E2 decreases proliferation signaling of human lung cancer cells. H460 cells were transduced with dE1-k35/LacZ or dE1-k35/sLRP6E1E2 (50 MOI) as described in Materials and Methods. The expression levels of MEK, Erk1/2, Survivin, mTOR, PI3K, and Akt was assessed by Western blot analysis.
(TIF)

Figure S4 sLRP6E1E2 decreases motility of H322 and H460 cancer cells. Cell migration was studied using a modified transwell migration chamber. (a) H322 and (b) H460 cells were transduced with dE1-k35/LacZ or dE1-k35/sLRP6E1E2 in the presence or absence of Wnt3a for 16 hr as described in Materials and Methods. Cells were then allowed to migrate for 20–24 hr. Migration was evaluated relative to untreated cells (100%). Assays were performed in triplicate and data shown are one representative experiment of three independent experiments performed. Results are reported as the mean ± SEM of 10 independent high power fields/well. ^#^ *P*<0.01, **P<0.001 versus PBS- or dE1-k35/LacZ-treated controls.
(TIF)

Figure S5 Decoy Wnt receptor sLRP6E1E2 inhibits epithelial-to-mesenchymal transition. Expression of EMT markers in H322 cells after 24 hr treatment with PBS, dE1-k35/LacZ, or dE1-k35/sLRP6E1E2 in the presence or absence of Wnt3a (100 ng/ml). Cells were stained with DAPI (blue), TRITC-labeled actin (red), or anti E-cadherin (green). Original magnification, ×400.
(TIF)
