# Supplementary figures and images for: A Novel sLRP6E1E2 Inhibits Canonical Wnt Signaling, Epithelial-to-Mesenchymal Transition, and Induces Mitochondria-Dependent Apoptosis in Lung Cancer
Source: PLoS One. 2012 May 14;7(5):e36520. doi: 10.1371/journal.pone.0036520 (PMC3351461; doi:10.1371/journal.pone.0036520)

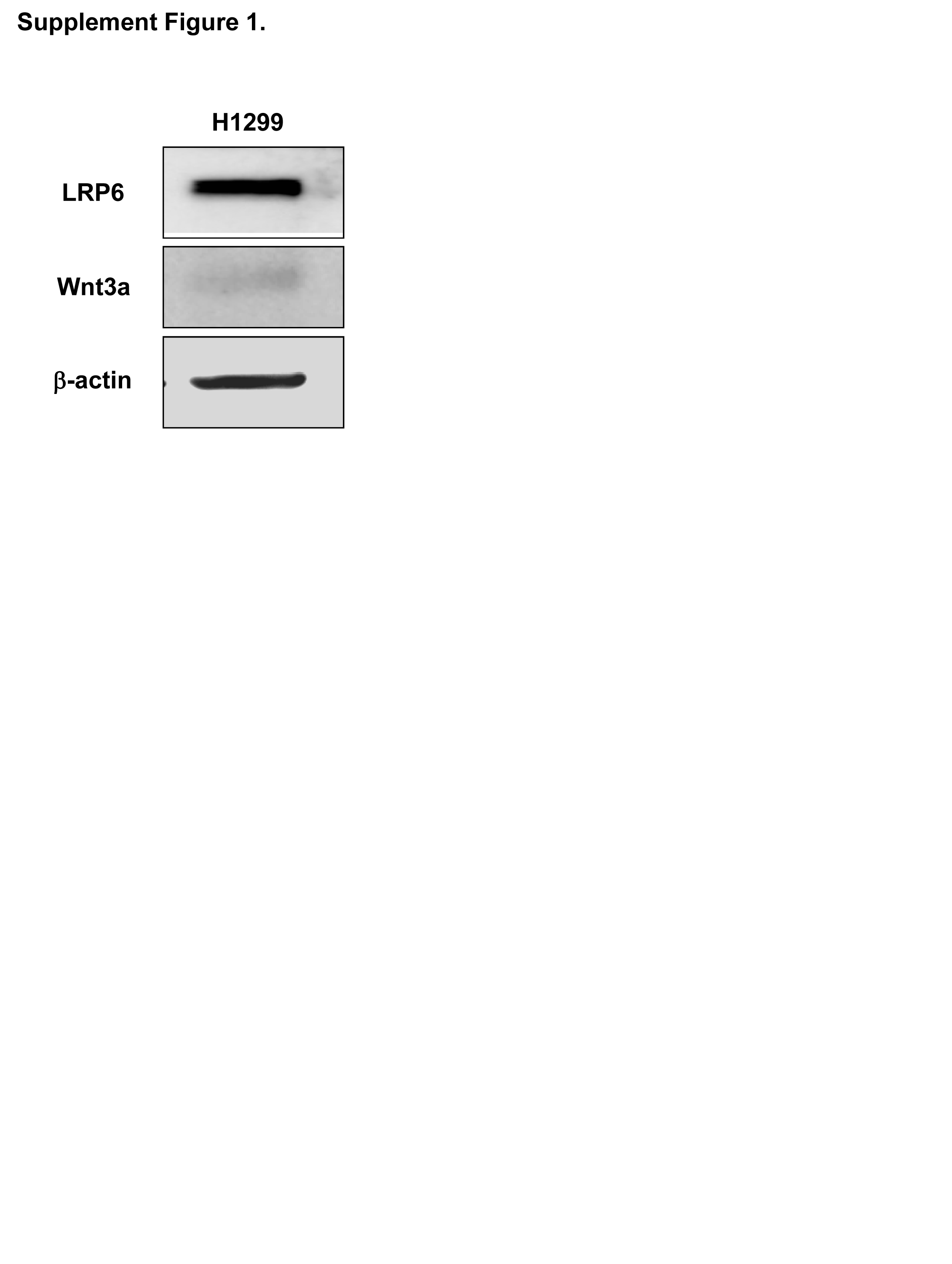

Supplement: Figure S1 — Endogenous LRP6 and Wnt3a expression in HT1299 human lung cancer cells. Western blot using antibodies specific to Wnt3a or LRP6. (TIF) [file pone.0036520.s001.tif]

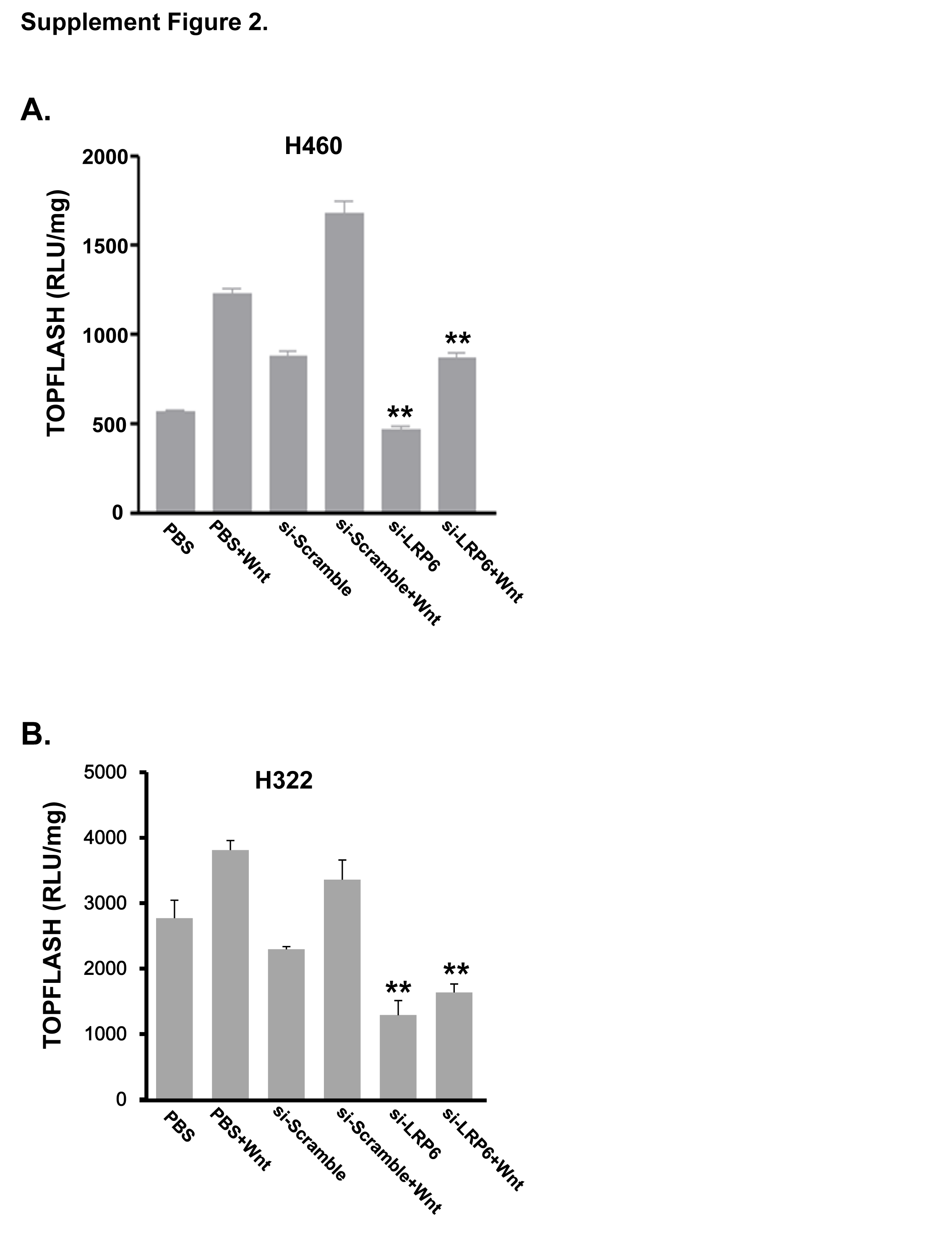

Supplement: Figure S2 — The effect of LPR6 knockdown on β-catenin/TCF transcriptional activity. H460 (a) and H322 (b) cells were co-transfected with TOPflash vector and LRP6 siRNA or control siRNA (si-Scramble) in the presence or absence of Wnt3a for 16 hr as described in Materials and Methods. **P<0.001 versus si-Scramble-transfected cells with or without Wnt3a. (TIF) [file pone.0036520.s002.tif]

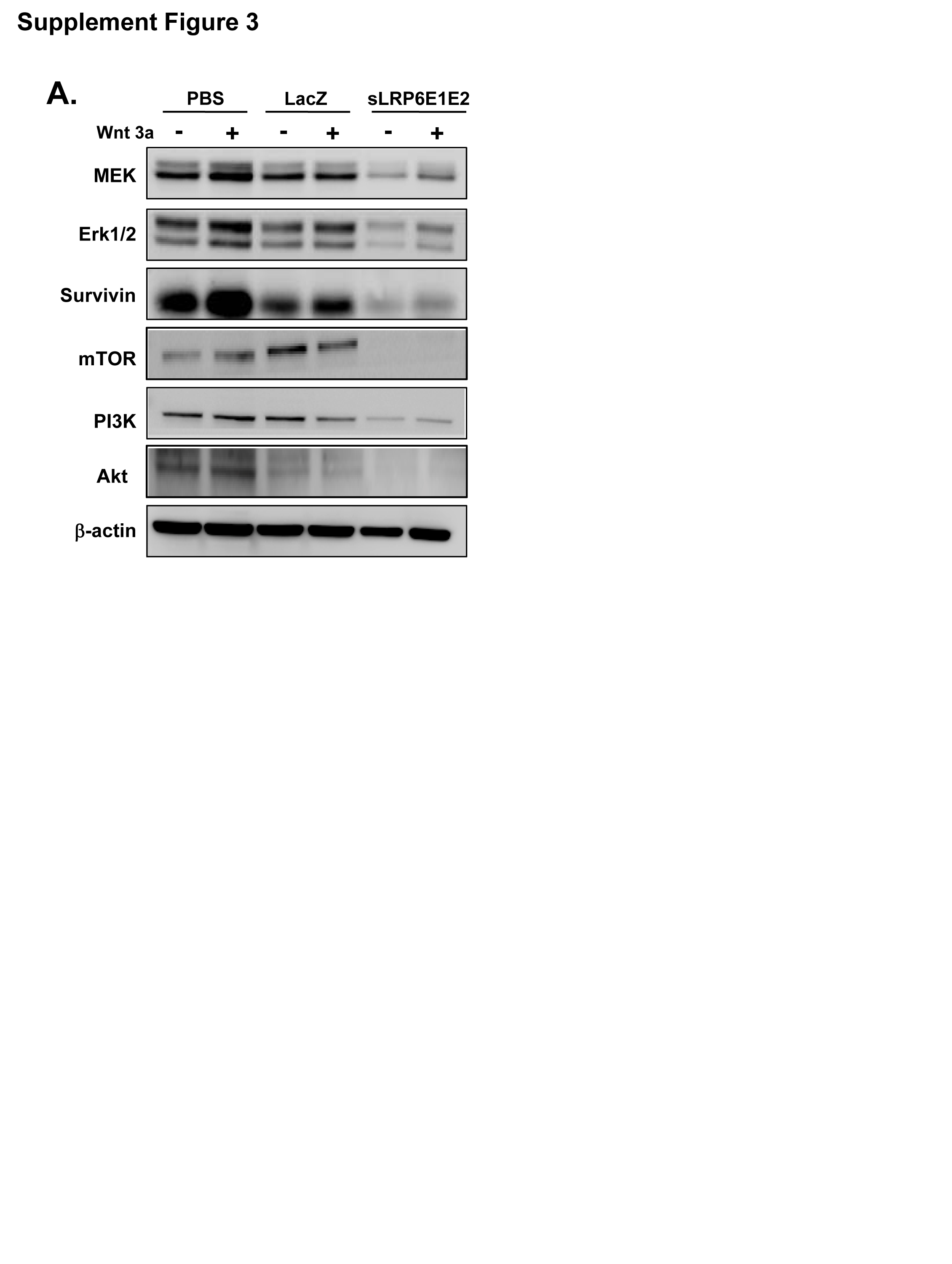

Supplement: Figure S3 — Decoy Wnt receptor sLRP6E1E2 decreases proliferation signaling of human lung cancer cells. H460 cells were transduced with dE1-k35/LacZ or dE1-k35/sLRP6E1E2 (50 MOI) as described in Materials and Methods. The expression levels of MEK, Erk1/2, Survivin, mTOR, PI3K, and Akt was assessed by Western blot analysis. (TIF) [file pone.0036520.s003.tif]

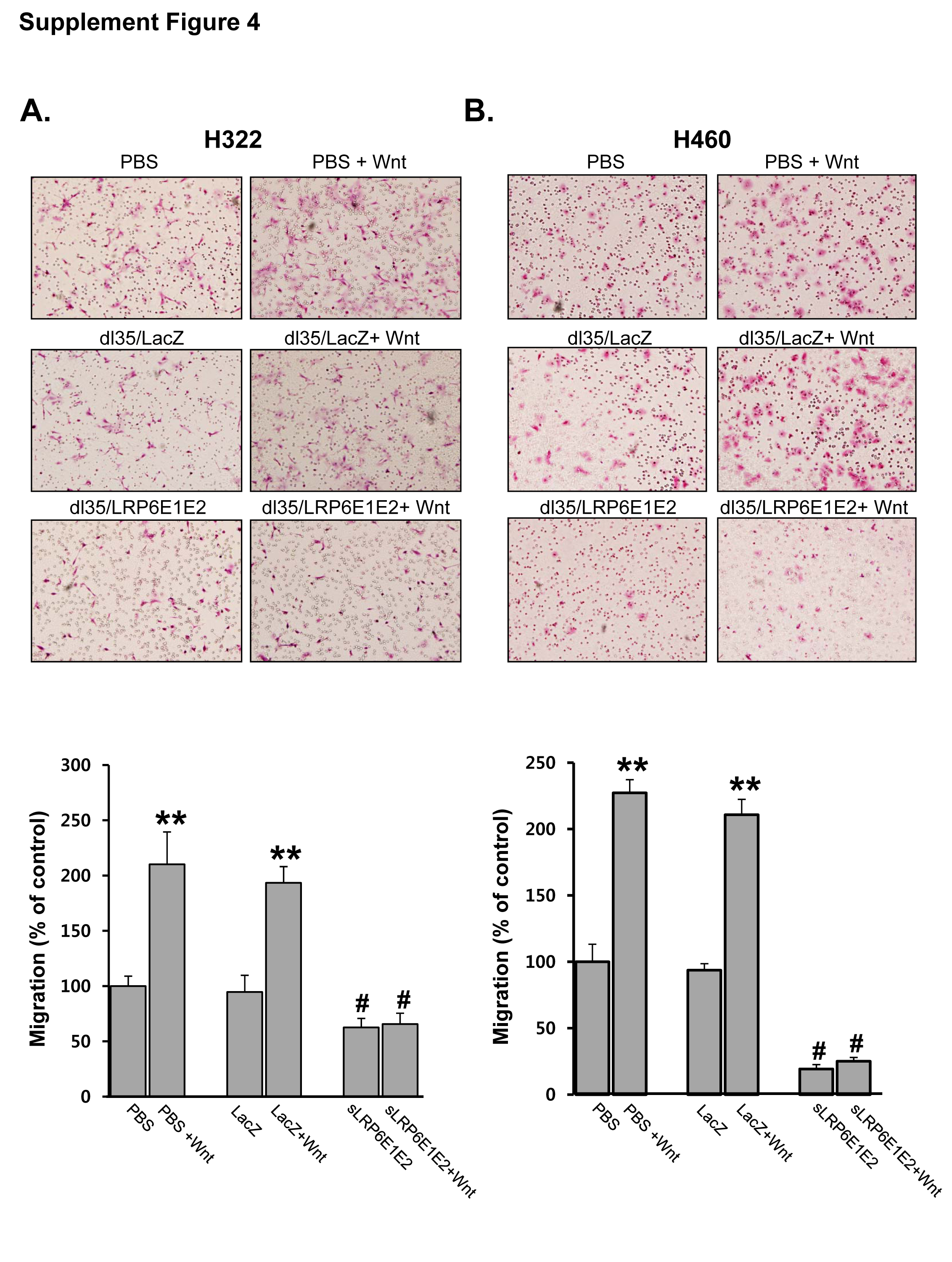

Supplement: Figure S4 — sLRP6E1E2 decreases motility of H322 and H460 cancer cells. Cell migration was studied using a modified transwell migration chamber. (a) H322 and (b) H460 cells were transduced with dE1-k35/LacZ or dE1-k35/sLRP6E1E2 in the presence or absence of Wnt3a for 16 hr as described in Materials and Methods. Cells were then allowed to migrate for 20–24 hr. Migration was evaluated relative to untreated cells (100%). Assays were performed in triplicate and data shown are one representative experiment of three independent experiments performed. Results are reported as the mean ± SEM of 10 independent high power fields/well. # P<0.01, **P<0.001 versus PBS- or dE1-k35/LacZ-treated controls. (TIF) [file pone.0036520.s004.tif]

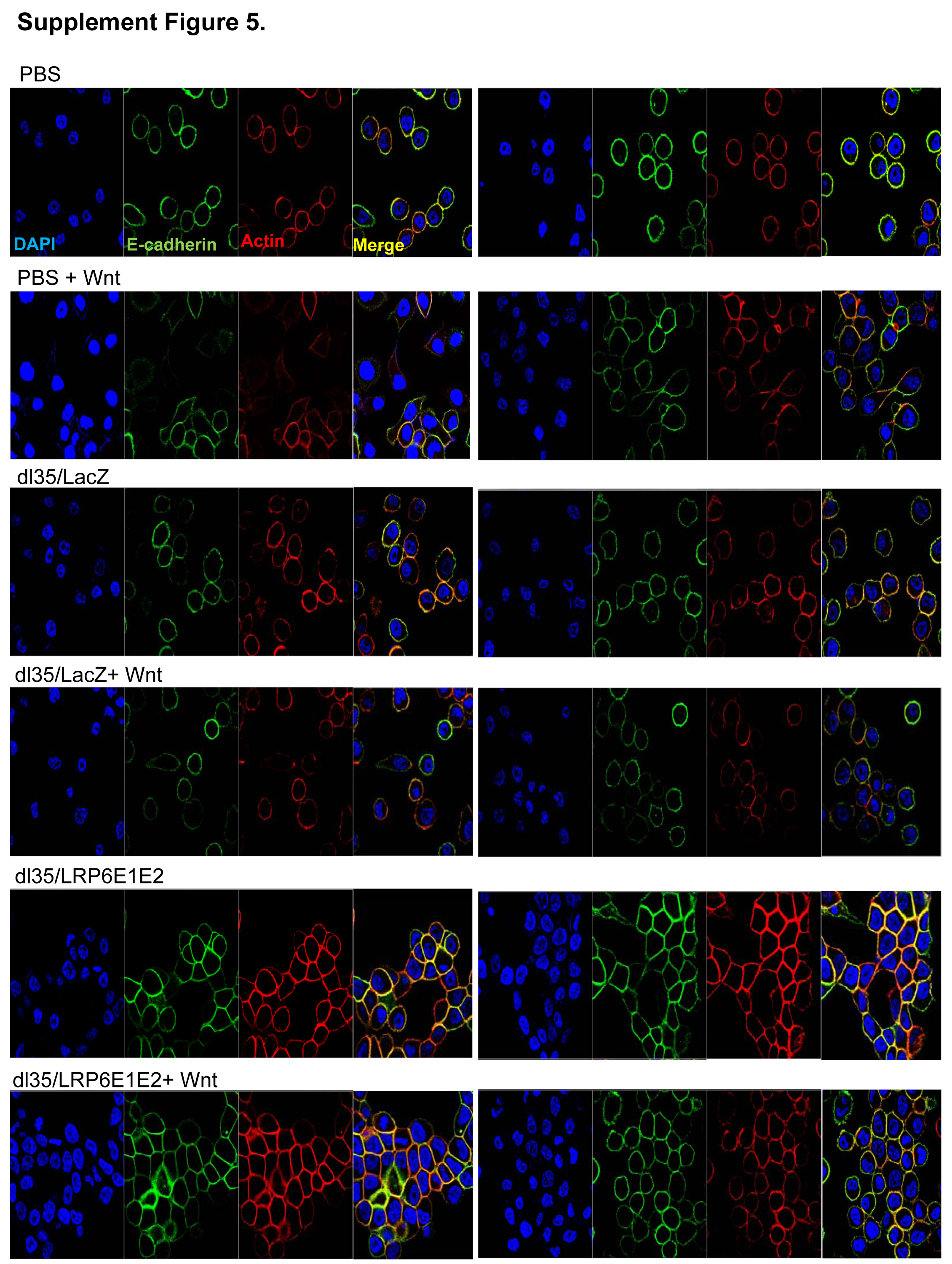

Supplement: Figure S5 — Decoy Wnt receptor sLRP6E1E2 inhibits epithelial-to-mesenchymal transition. Expression of EMT markers in H322 cells after 24 hr treatment with PBS, dE1-k35/LacZ, or dE1-k35/sLRP6E1E2 in the presence or absence of Wnt3a (100 ng/ml). Cells were stained with DAPI (blue), TRITC-labeled actin (red), or anti E-cadherin (green). Original magnification, ×400. (TIF) [file pone.0036520.s005.tif]
